# CbRCI35, a Cold Responsive Peroxidase from *Capsella bursa-pastoris* Regulates Reactive Oxygen Species Homeostasis and Enhances Cold Tolerance in Tobacco

**DOI:** 10.3389/fpls.2016.01599

**Published:** 2016-10-21

**Authors:** Mingqi Zhou, Weiwei Li, Ye Zheng, Ping Lin, Xiaohua Yao, Juan Lin

**Affiliations:** ^1^State Key Laboratory of Genetic Engineering, Institute of Plant Biology, School of Life Sciences, Fudan UniversityShanghai, China; ^2^Research Institute of Subtropical Forestry, Chinese Academy of ForestryFuyang, China

**Keywords:** *CbRCI35*, cold tolerance, gene expression, ROS, tobacco

## Abstract

Low temperature affects gene regulatory networks and alters cellular metabolism to inhibit plant growth. Peroxidases are widely distributed in plants and play a large role in adjusting and controlling reactive oxygen species (ROS) homeostasis in response to abiotic stresses such as low temperature. The Rare Cold-Inducible 35 gene from *Capsella bursa-pastoris* (*CbRCI35*) belongs to the type III peroxidase family and has been reported to be a cold responsive gene in plants. Here we performed an expressional characterization of *CbRCI35* under cold and ionic liquid treatments. The promoter of *CbRCI35* was also cloned and its activity was examined using the GUS reporter system. CbRCI35 protein was localized in the cytoplasm according to sequence prediction and GFP fusion assay. Heterologous expression tests revealed that *CbRCI35* conferred enhanced resistance to low temperature and activated endogenous cold responsive signaling in tobacco. Furthermore, in the normal condition the ROS accumulation was moderately enhanced while after chilling exposure superoxide dismutase activity was increased in *CbRCI53* transgenic plants. The ROS metabolism related genes expression was altered accordingly. We conclude that *CbRCI35* modulates ROS homeostasis and contributes to cold tolerance in plants.

## Introduction

Low temperature impacts plant development as well as propagation, and imposes restriction on plant distribution (Thomashow, 1999; Beike et al., 2015). In the face of cold stress, plants reorganize gene regulatory networks and adjust cellular metabolic reactions to gain enhanced cold resistance (Narusaka et al., 2004; Yue et al., 2015). The ability of plants to tolerate environmental stresses is highly associated with signaling molecules (Mengel et al., 2013). Reactive oxygen species (ROS) is one of the key signals regulating plant bioprocesses through the activation of secondary messengers, the gene transcription switch and the enzyme activity variation (Lamotte et al., 2014; Farnese et al., 2016). Biosynthesis and metabolism of ROS such as hydrogen peroxide, superoxide, singlet oxygen and hydroxyl radicals influence the redox state of plant cells in the early stages of stress response (Mittler, 2002; Del Rio, 2015). During the cold response, a burst of ROS following a metabolic imbalance of energy appears to elicit cellular damage and abnormity (Potters et al., 2007). On the other hand, the rapidly accumulated ROS mediates stress responsive signaling transduction and functions in an essential manner for cold acclimation (Zhao et al., 2009; Hossain et al., 2015). In fact, low doses of NO or H_2_O_2_ contribute to cold hardiness in multiple plant species such as *Arabidopsis* *thaliana* and *Brassica juncea* (Neill et al., 2002; Abat and Deswal, 2009; Cantrel et al., 2011). It has been reported that non-toxic levels of H_2_O_2_ accumulation are necessary in the plant acclimation of various stresses including low temperature (Petrov and Van Breusegem, 2012). The maintained proper concentration of H_2_O_2_ plays a role in the delicate balancing between H_2_O_2_ production and scavenging during stress response (Hossain et al., 2015). Moderately elevated ROS can activate cold responsive gene expression and mediate protective mechanisms against damage to macromolecules and cell structures in cold acclimation of plants (Yu et al., 2003; Hung et al., 2007; Wang et al., 2010). In tomato it has been documented that H_2_O_2_ significantly strengthens antioxidant process to reduce oxidative damage (Iseri et al., 2013). Given all the clearly presented evidence showing the crucial role of ROS as a signaling molecule in cold response, information in regards to the function of ROS regulators in stress response needs to be enriched.

Plant cells possess a protective system that is comprised of enzymatic and non-enzymatic antioxidants against oxidative stress (Mittler et al., 2004). Peroxidases such as catalase (CAT) and ascorbate peroxidase (APX) act as key enzymes in modulating ROS homeostasis in response to environmental stimulus or developmental transitions (Navabpour et al., 2003). Peroxidases are widely distributed throughout the plant kingdom (Mishra et al., 2016). The type III peroxidase is a superfamily of plant-specific heme oxidoreductases that can be encoded by a large number of genes (Kvaratskhelia et al., 1997; Shigeto and Tsutsumi, 2016). In *Arabidopsis*, 73 type III peroxidase genes have been identified and they are implicated in a widely diverse range of bioprocesses (Tognolli et al., 2002). For example, type III peroxidases either augment or reduce the apoplastic ROS levels during cell wall development (Mangano et al., 2016). Among these genes the Rare Cold-Inducible 3 (*AtRCI3*) was isolated through a screening using a cDNA library from etiolated *Arabidopsis* plants after cold acclimation (Llorente et al., 2002). *AtRCI3* showed a strong cold-induced expression pattern and encoded an active cationic peroxidase. Further analysis revealed that *AtRCI3* enhanced the ROS production under potassium deficiency (Kim et al., 2010). Although identification of *AtRCI3* is conducted by screening in cold responsive transcripts, studies exploring the molecular function of *AtRCI3* or *AtRCI3*-like genes in cold response are limited.

The Rare cold-inducible 35 gene (*CbRCI35*) is cloned from *Capsella bursa-pastoris* that is grown in temperate regions but has a strong ability to tolerate low temperature (Lin et al., 2007). The *CbRCI35* gene encodes a cold-inducible protein that is highly homologous to AtRCI3. In the present work, the expressional analysis and promoter activity test of *CbRCI35* under cold temperatures, plant hormone and ionic treatments were performed. Functional characterization of transgenic tobacco was also performed to show that *CbRCI35* regulates ROS homeostasis and cold tolerance of plants.

## Materials and Methods

### Plant Materials and Treatments

The seeds of *C. bursa-pastoris* and *Nicotiana tabacum* were stored as previously described (Zhou et al., 2012). The seeds of *A. thaliana* of Columbia (Col-0) accession were obtained from the *Arabidopsis* Biological Resource Center (ABRI: Columbus, OH, USA). Plants were grown at stable temperature (22°C) and light conditions (16 h light/8 h dark). For cold application of *C. bursa-pastoris*, the 4-week-old plants were subjected to 4°C for 4, 8, or 24 h. Meanwhile the seedlings of the same age were shifted from 22 to 12°C for 4 days, 4°C for 4 days, 0°C for 2 h continuously for cold acclimation. The roots, stems and leaves were collected at each time point and frozen in liquid nitrogen immediately. For ionic liquid treatments, 2-week-old *C. bursa-pastoris* seedlings were soaked with 80 mM KCl, 50 mM MgCl_2_, 5 mM ZnCl_2_, 30 mM LiCl, 0.1 mM CuCl_2_, 80 mM CaCl_2_ solution for 0, 4, 8, and 24 h. For phytohormone treatments, 2-week-old *A. thaliana* plants were soaked with 5 μM gibberellin (GA), 300 μM methyl jasmonate (MeJA), and 500 μM salicylic acid (SA) solution for 0, 1, 6, and 24 h, respectively. The whole seedlings were harvested for RNA extraction. For the cold tolerance test of *N. tabacum*, 4-week-old tobacco plants were subjected to 4°C for 24 h, -4°C for 1 h and 22°C for 2 days in turn.

### Cloning of *CbRCI35* Promoter from *C. bursa-pastoris*

The genomic DNA of *C. bursa-pastoris* is obtained by CTAB extraction method. According to the manual of the Universal Genome Walker TM Kit (CLONTECH), the genome walker library was constructed and nested amplification was conducted using specific primers RCI35GSP1 and RCI35GSP2 paired with the adaptor primers AP1 and AP2. A 1011 bp of 5′ upstream sequence of *CbRCI35* was cloned and analyzed by PLANTCARE^1^ (Zhou et al., 2014).

### Plant Transformation

For characterization of promoter activity, *CbRCI35* promoter clones were constructed in the *Kpn*I/*Nco*I site of the pCAMBIA1301 vector (CAMBIA, Australia) with primers pCbRCI35-F and pCbRCI35-R. The pCbRCI35::GUS plasmid was transformed into *Arabidopsis* of Col-0 plants with the Agrobacterium-mediated floral dip method. The T1 plants were selected on hygromycin (30 mg/L) media and further confirmed by PCR. The T2 lines showing 3:1 segregation were carried forward to the T3 generation. PCR positive homozygous T3 and T4 lines were used for further analyses of the *CbRCI35* promoter.

For gene overexpression, *CbRCI35* mRNA sequence information was previously described (Lin et al., 2007). The *CbRCI35* cDNA clones were constructed in the *Nco*I/*Nhe*I site of the pCAMBIA1304 vector (CAMBIA, Australia) with primers CbRCI35-nco-F and CbRCI35-nhe-R, using hygromycin resistance as a selection marker. The 35S::CbRCI35 plasmid was introduced into leaf disks of tobacco using Agrobacterium EHA105. Plants transformed with the empty vector were used as a blank control. Transformants were selected on MS medium solidified with 0.8% agar containing 0.1 mg/L NAA, 1.0 mg/L 6-BA, 250 mg/L carbenicillin disodium and 30 mg/L hygromycin, and regenerated on hormone-free MS medium containing 250 mg/L carbenicillin disodium at 22°C. Transformants were identified by DNA-PCR analysis with primers Hyg-F and Hyg-R (**Table 1**).

**Table 1:** Primers used in this work.

Name	Sequence
CbRCI-F	5′- ATGAACTGCTTGAGAGCTATTG-3′
CbRCI-R	5′- ACTATTTGCAACGGAACATTGC-3′
RCI35GSP1	5′-CTCGCACGAAACAATCATGGAAATGC-3′
RCI35GSP2	5′-CGTTAGGACAAGTATTGGCATAGAAGT-3′
AP1	5′- GTAATACGACTCACTATAGGGCGTAATACGACTCACTATAGGGC-3′
AP2	5′- ACTATAGGGCACGCGTGGT-3′
pCbRCI35-F	5′-GggtaccCTGCTCAACTACCACTAATTC-3′ (Kpn I site is labeled)
pCbRCI35-R	5′-TccatggCGTTGTGGGGGTTTTTTTTTC-3′ (Nco I site is labeled)
CbRCI35-nco-F	5′-GCccatggTAATGAACTGCTTGAGAGCTATTG-3′ (Nco I site is labeled)
CbRCI35-bgl-R	5′-GAagatctTCACTATTTGCAACGGAACATTGC-3′ (Bgl II site is labeled)
CbRCI35-nhe-R	5′-CTAgctagcTAGACTATTTGCAACGGAACATTGC-3′ (Nhe I site is labeled)
Hyg-F	5′-GTCGAGAAGTTTCTGATCG-3′
Hyg-R	5′-GTTTCCACTATCGGCGAGTACT-3′
GUS-F	5′-GCTCTACACCACGCCGAACACCTG-3′
GUS-R	5′-TCTTCAGCGTAAGGGTAATGCGAGGTA-3′
Realtime-CbRCI35-F	5′- CATTAGCCAACATTCCTCCTCCGACCA-3′
Realtime-CbRCI35-R	5′- CTGACTGAAACAGACCTCTACGCTTG-3′
Realtime-CbActin-F	5′- ATGCTCCCAGGGCTGTTTTC-3′
Realtime-CbActin-R	5′- TTCCATATCGTCCCAGTTGC-3′
Realtime-NtDREB1-F	5′- CAGGTAAGTGGGTGTGTGAAGTG-3′
Realtime-NtDREB1-R	5′- TGCGATCTCGGCTGTTAGG-3′
Realtime-NtDREB3-F	5′- TACAGGGGAGTGAGGAAGAGGA-3′
Realtime-NtDREB3-R	5′- GCAGAAGGGAAAGTGCCAAG-3′
Realtime-NtERD10a-F	5′- TGAGAAGAAGGGAATTATGGACAAG-3′
Realtime-NtERD10a-R	5′- CGCAGCAGATTTTCTAGTGGTG-3′
Realtime-NtERD10b-F	5′- ATCACACTGGAGGTACCATGGG-3′
Realtime-NtERD10b-R	5′- CTTCTTCCTTCTTCCGCCTTG-3′
Realtime-NtAPX-F	5′- CAAATGTAAGAGGAAACTCAGAGGA-3′
Realtime- NtAPX-R	5′- CAGCCTTGAGCCTCATGGTACCG-3′
Realtime- NtCAT-F	5′- AGGTACCGCTCATTCACACC-3′
Realtime- NtCAT-R	5′- AAGCAAGCTTTTGACCCAGA-3′
Realtime- NtGST-F	5′- CCCCTAGTTTGCTCCCTTCT-3′
Realtime- NtGST-R	5′- TTCTTAGCTGCCTCCTGCTC-3′
Realtime- NtSOD-F	5′- CTCCTACCGTCGCCAAAT-3′
Realtime- NtSOD-R	5′- GCCCAACCAAGAGAACCC-3′
Realtime- NtRBOHD1-F	5′- CAAATGTAAGAGGAAACTCAGAGGA-3′
Realtime- NtRBOHD1-R	5′- GTACACAATAGGGAGAGTTGGTAGAC-3′
Realtime- NtRBOHD2-F	5′- AGATACCAAGGGAATTAAGAATGTG-3′
Realtime- NtRBOHD2-R	5′- GGCACCCATCAAAAGAGG-3′
Realtime-NtActin-F	5′- GGAAAGTCCTACCAGCATTG-3′
Realtime-NtActin-R	5′-ATCTATTGTCTCCCACGAAG-3′
Realtime-GUS-F	5′-GCTCTACACCACGCCGAACACCTG-3′
Realtime-GUS-R	5′-TCTTCAGCGTAAGGGTAATGCGAGGTA-3′
Realtime-actin2-F	5′- TGAGAGATTCAGATGCCCAGAA-3′
Realtime-actin2-R	5′- TGGATTCCAGCAGCTTCCAT-3′


### Histochemical GUS Staining and Semi-Thin Section

The seedlings of 2.5-week-old transgenic *Arabidopsis* with pCbRCI35::GUS at 22°C or after 4°C treatment for 8 h were collected and soaked in GUS staining buffer containing 0.075% X-Gluc (5-bromo-4-chloro-3-indolyl-bd-glucuronic acid), 3 mM potassium ferricyanide, 7.2 mM EDTA, 57.7 mM disodium phosphate, 42.3 mM sodium phosphate, and 0.005% Triton X-100 followed by vacuum infiltration for 20 min. Afterward the seedling samples were incubated at 37°C overnight and de-stained in 75% ethanol at 60°C for several times until chlorophyll was removed. Another set of 4.5-week-old transgenic plants were stained and embedded with Technovit 7100 plastic embedding kit (Heraeus Kulzer, Germany) following the manufacturer’s instructions and semi-sectioned by Leica 2265 Rotary Microtome (Leica, Germany). The sections were observed with Zeiss Scope A1 microscope (Zeiss, Germany).

### Subcellular Localization of CbRCI35

The *CbRCI35* cDNA clones were constructed in the *Nco*I/*Bgl*II site of the pCAMBIA1302 vector (CAMBIA, Australia) with primers CbRCI35-nco-F and CbRCI35-bgl-R. The onion epidermis discs were incubated with a culture of Agrobacterium carrying the 35S::CbRCI35-GFP plasmid for 20 min. Acetosyringone (100 μM) was added to enhance the transformation efficiency. After grown in the dark at 25°C for 2 days, the GFP signal was detected by confocal laser scanning microscopy (Zeiss 710, Germany) and the images were analyzed with Zen software.

### Quantitative Real-Time PCR

Quantitative real-time PCR (qPCR) was employed to analyze gene relative expression levels. The total RNA was extracted using Plant RNA Mini Kit (CW Biotech Inc., Ltd, China) and the cDNA was synthesized using PrimeScript^®^ RT Master Mix (TaKaRa, China) according to the manufacturer’s instructions. The qPCR was carried out using SYBR^®^ Premix Ex Taq^TM^ II (Perfect Real-Time; TaKaRa, China) on a StepOnePlus^TM^ Real-Time PCR System (Applied Biosystems) with three replicates. The PCR procedure was 95°C for 30 s, 40 cycles of 95°C for 5 s, and 60°C for 34 s, followed by 95°C for 15 s, 60°C for 1 min, and 95°C for 15 s. The *CbACTIN* (HQ880662), *ACTIN2* (AK230311) and *NtACTIN* (AJ133422) were used as the internal control for *C. bursa-pastoris. A. thaliana*, and *N. tabacum*, respectively.

### Physiological Indices Measurement

Physiological indices were measured as previously described (Wu et al., 2012). In brief, leaves of tobacco were incubated in 2 ml deionized water at room temperature for 12 h and the sample conductivities (C1) were measured with a DDS-11A meter (Shanghai SUOSHEN Electrical Equipment Co. Ltd., China). Then the samples were boiled in deionized water at 100°C for 1 h and cooled to room temperature to get the sample conductivities (C2). Relative electrolyte leakage was calculated using the following formula: C1/C2 × 100%. Relative water content (%) was calculated as 100% × (FW-DW)/(TW-DW), in which the fresh weight (FW) and the turgid weight (TW) were determined before and after leaves dipped in 4 ml deionized water for 12 h at room temperature, while the dry weights (DW) were measured after samples were oven dried at 65°C for 24 h. Glucose content was assayed by measuring the NADH production using the Glucose (HK) Assay kit (Sigma–Aldrich, Inc.) depending on absorbance at 340 nm (*A*_340_). The H_2_O_2_ content was measured according to *A*_415_ of the titanium-peroxide complex as previously described (Jiang and Zhang, 2001). The superoxide dismutase (SOD) activity was determined by an inhibited photoreduction rate of nitro blue tetrazolium (NBT) (Mckersie et al., 1993). The malondialdehyde (MDA) content was measured using the thiobarbituric acid (TBA)-based colorimetric method as reported previously (Draper et al., 1993). The BioPhotometer Plus (Eppendorf, Germany) was used for electromagnetic absorbance reading in each assay. Three biological replicates were used for each experiment and the data were analyzed by Student’s *t*-test.

### Dichloro-Dihydro-Fluorescein Diacetate Assay

Tobacco leaf disk samples were immersed with 10 μM carboxy-2′,7′-dichloro- dihydro-fluorescein diacetate (DCFH-DA) probe at 37°C for 20 min in the dark (Ezaki et al., 2000). Samples were subsequently washed with 20 mM potassium phosphate buffer (pH 6.0) for three times to remove extra probe. Fluorescent signals were visualized using a confocal laser scanning microscope (Zeiss 710, Germany).

## Results

### Expression Pattern of *CbRCI35* under Cold and Ionic Treatments

**FIGURE 1:**
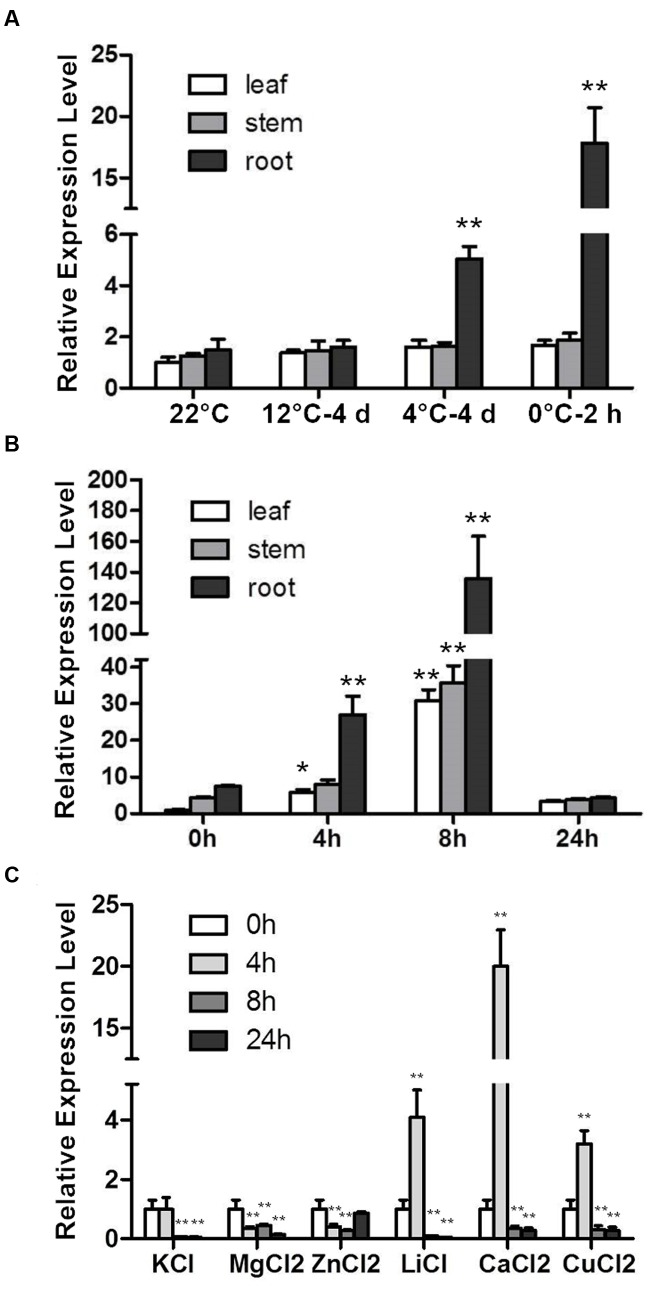
**Induction of *CbRCI35* in *Capsella bursa-pastoris* plants under different treatments.** **(A)** The *CbRCI35* expression was analyzed in leaves, roots and stem in 4-week-old plants growing at 22°C after shifting to 12°C for 4 days, 4°C for 4 days and 0°C for 2 h in turn. **(B)** The *CbRCI35* expression in leaves, roots and stem in 4-week-old plants growing at 22°C was tested after treatments at 4°C for 0, 4, 8, and 24 h. **(C)** The *CbRCI35* expression was examined in 2-week-old seedlings after treatments with different proton solutions at normal temperature for 0, 4, 8, and 24 h. *CbActin* gene was used as a housekeeping control. Expression levels were relative to control condition in each tissue. Data are means ± SD (*n* = 3, ^∗^*P* < 0.05, ^∗∗^*P* < 0.01).

The *RCI* genes were identified according to their cold-induced expression pattern. *CbRCI35* was responsive to cold temperatures in young *C. bursa-pastoris* plants (Lin et al., 2007). For a more detailed characterization, the expression patterns of *CbRCI35* in different tissues in response to low temperature were detected in a cold acclimation assay and a time course assay. *CbRCI35* showed a root-specific and temperature-dependent expression pattern in *C. bursa-pastoris* during cold acclimation from 12, 4 to 0°C. After 4 days of 12°C application, the *CbRCI35* transcript did not change in leaves, stems or roots compared with the 22°C control. When treated at 4°C for 4 days followed by 0°C for 2 h, significant enhancements were observed only in roots and 0°C activated the highest observed transcription level (**Figure 1A**). In a time course assay, the basal level of *CbRCI35* mRNA was highest in roots, and was lower in stems and leaves. During cold application, *CbRCI35* transcription was elevated at the time point of 4 h and reached its peak after 8 h in 4°C. As before, in roots it showed the highest expression level, while in leaves and stems the gene expression pattern had a similar trend but the transcript levels were lower (**Figure 1B**).

The RCI genes coding peroxidases such as *AtRCI3* are involved in K^+^ transportation (Kim et al., 2010) and the protein activity can be affected by ion concentration (Lai et al., 2006). We detected *CbRCI35* expression in response to 80 mM KCl, 50 mM MgCl_2_, 5 mM ZnCl_2_, 30 mM LiCl, 0.1 mM CuCl_2_, 80 mM CaCl_2_ in young seedlings of *C. bursa-pastoris*. Consistent with the induced transcription of *AtRCI3* under K^+^ deficiency, *CbRCI35* expression was repressed by K^+^ application. Mg^2+^ also down-regulated *CbRCI35* expression and Zn^2+^ caused a transient suppression. For Li^+^, Cu^2+^, and Ca^2+^, the transcription of *CbRCI35* showed a significant transient elevation in the early stage, suggesting that *CbRCI35* might play a role in Li^+^, Cu^2+^, and Ca^2+^ transportation (**Figure 1C**).

### Cloning and Characterization of *CbRCI35* Promoter from *C. bursa-pastoris*

**FIGURE 2:**
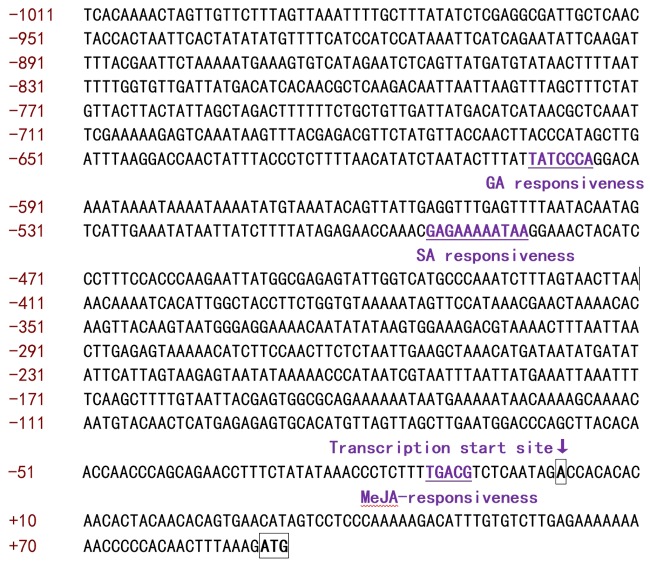
**Sequences of the 5′ upstream flanking region of *CbRCI35*.** Numbering is relative to the transcription start site which was designated +1 and indicated with an arrow. gibberellin (GA), salicylic acid (SA), and methyl jasmonate (MeJA) responsiveness associated elements were labeled and underlined.

**FIGURE 3:**
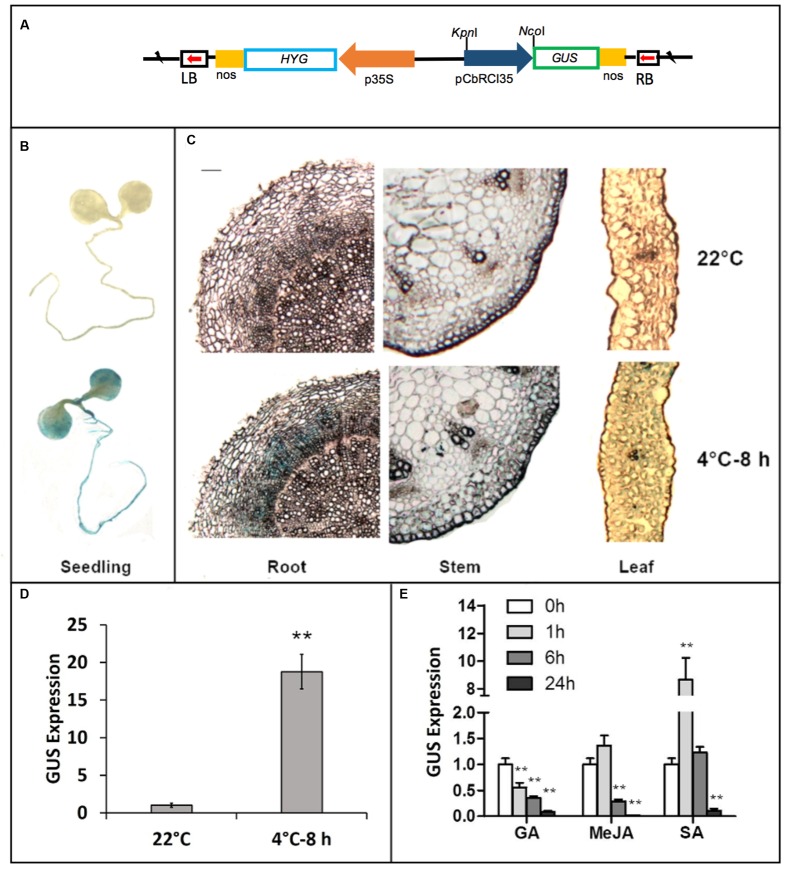
**Characterization of *CbRCI35* promoter activity using GUS report system and qPCR.** **(A)** The GUS reporter gene was under the control of the *CbRCI35* promoter in pCbRCI35::GUS plasmid. **(B)** GUS staining of 2.5-week-old transgenic *Arabidopsis* seedlings containing pCbRCI35::GUS fusion growing at 22°C or exposed to 4°C for 8 h. **(C)** Semi-thin section of leaf, stem and root tissues from 4.5-week-old transgenic seedlings containing pCbCOR15a::GUS fusion growing at 22°C or exposed to 4°C for 8 h after GUS staining. The bar represents 0.001 cm. Transcript level of GUS reporter gene driven by CbRCI35 in transgenic plants growing at 22°C was tested after exposed to 4°C for 8 h **(D)** or treated by GA, MeJA, or SA for time indicated **(E)**. *Actin2* gene was performed as an internal control. Data are means ± SD (*n* = 3, ^∗∗^*P* < 0.01).

A fragment of 1011 bp in the promoter region of *CbRCI35* was isolated from the *C. bursa-pastoris* genome by DNA walking (**Figure 2**). Sequence analysis was performed using the PLANTCARE software^2^. Three *cis*-acting elements involved in gibberellin (GA), SA and MeJA responsiveness were identified, respectively. To further characterize the *CbRCI35* promoter, a GUS reporter system driven by the 1011 bp *pCbRCI35* promoter fragment was created (**Figure 3A**). Histochemical GUS staining indicated extremely low levels of GUS activity throughout the entire seedling at 22°C, and increased activity after 8 h exposure to 4°C in 2-week-old plants (**Figure 3B**). Correspondingly, a markedly increased level of GUS mRNA in response to cold was detected in 2-week-old seedlings (**Figure 3C**). For a more accurate, tissue-specific analysis, semi-thin sections of 4.5-week-old plant organs were dissected out after staining. In contrast to what is observed in younger seedlings, GUS accumulation was mainly in the roots, while comparatively lower GUS enhancement was observed in stems and leaves (**Figure 3D**). The GUS activity in roots was displayed in the cortex but not the epidermal or vascular tissues, implying the protective function of CbRCI35 for the cortical cells containing stored carbohydrates and other substances. These indicated that the cold-induced activity of *CbRCI35* was in an age-dependent, tissue-specific manner. Considering the predicted *cis*-acting elements in the *CbRCI35* promoter, phytohormone responsive activity was subsequently investigated using GA, SA, and MeJA treatments. Interestingly, GA and MeJA inhibited *CbRCI35* transcription while SA conferred transient upregulation of gene expression in early stages and downregulation after 24 h of application (**Figure 3E**). The promoter characterization revealed a cold and phytohormone inducible expression pattern of *CbRCI35*.

### Subcellular Localization of CbRCI35 Protein

**FIGURE 4:**
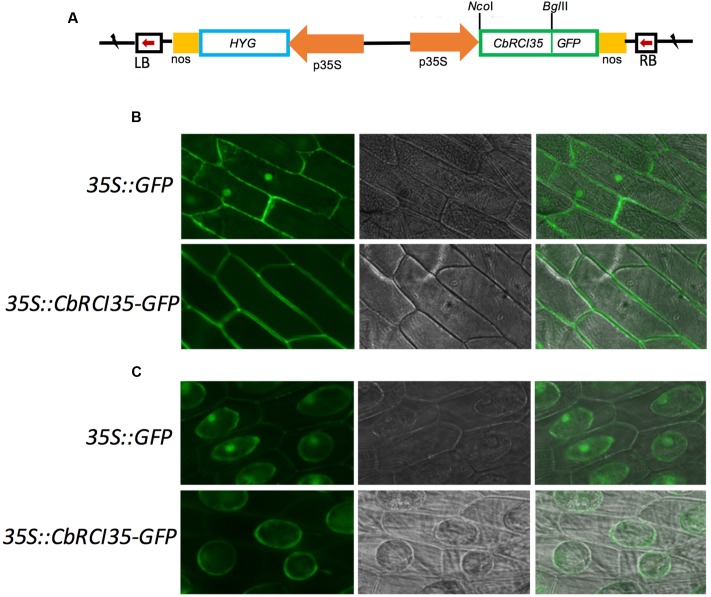
**Subcellular localization of CbRCI35 protein.** **(A)** The structure of 35S::CbRCI35-GFP fusion plasmid. **(B)** Subcellular localization of CbRCI35 in onion epidermis cells. **(C)** GFP signal in onion epidermis cells after plasmolysis in 2 g/ml sucrose solution. Photographs of cells expressing CbRCI35-GFP fusion protein and GFP only were taken in bright light to observe cell morphology (middle) and in dark-field to observe green fluorescence (left). The right ones are merged pictures.

Given the tissue-specific expression pattern of *CbRCI35*, it was relevant to study the subcellular localization of the CbRCI35 protein to further clarify its function. It was reported that AtRCI3, a highly homologous protein to CbRCI35, was a secreted protein and could be sorted to the cell wall (Kim et al., 2010). The online tool Plant-mPLoc^3^ suggested that CbRCI35 was localized in the cytoplasm. Using a transient expression assay, CbRCI35-GFP fusion protein driven by the 35S promoter was expressed in onion epidermal cells (**Figure 4A**). The CbRCI35-GFP promoter was distributed around the periphery of onion cells, while the signal of the GFP control was also detected in the nucleus (**Figure 4B**). After plasmolysis in 2 g/mL sucrose solution, the CbRCI35-GFP fusion signal was observed in the cytoplasm but not the cell wall (**Figure 4C**). These showed that unlike secretion of AtRCI3, CbRCI35 is localized in the cytoplasm, although the sequence similarity of these two proteins is as high as 95% (Lin et al., 2007).

### Overexpression of *CbRCI35* Enhanced Cold Tolerance in Tobacco

**FIGURE 5:**
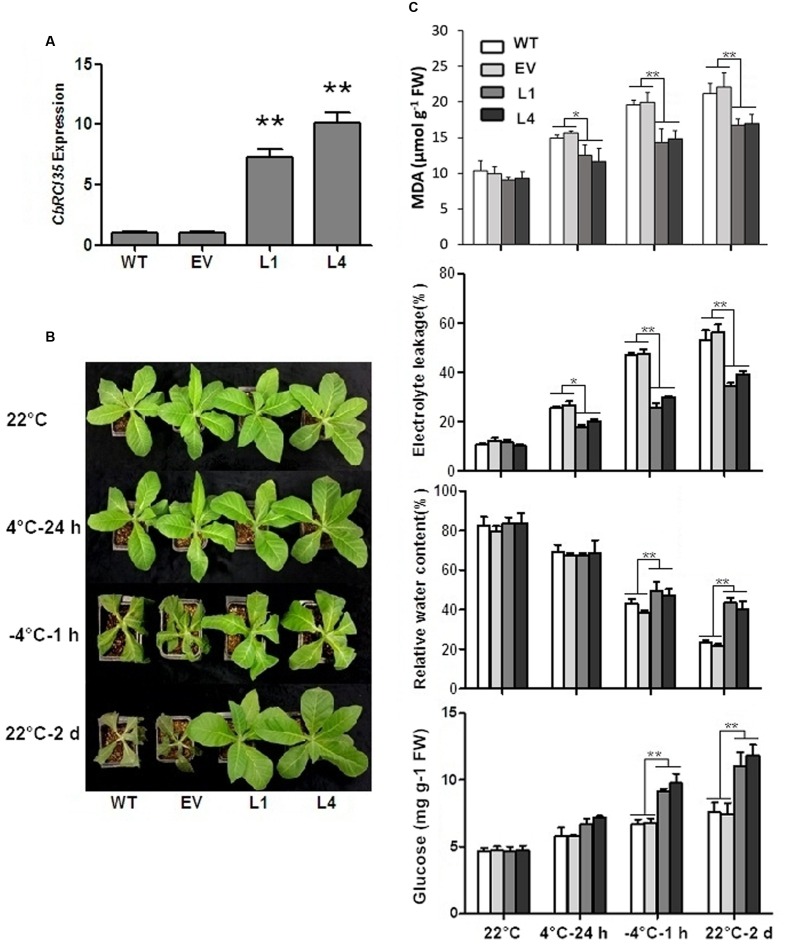
***CbRCI35* enhanced cold tolerance of tobacco plants.** **(A)** The relative expression of *CbRCI35* in transgenic tobacco plants. The *NtActin* gene was performed as an internal control. **(B)** Wild type (WT), empty vector control (EV) and *CbRCI35-ox* lines L1, L2 were grown at 22°C. After treated at 4°C for 24 h, at -4°C for 1 h, and at 22°C for a 2-day recovery, plants phenotypes were shown. They were representatives of their respective types of tobacco plants. **(C)** The malondialdehyde (MDA) content, electrolyte leakage, relative water content and glucose content of tobacco leaves before and after cold application. Error bars indicate SD (*n* = 3, ^∗^*P* < 0.05, ^∗∗^*P* < 0.01).

Next, the functional analysis of *CbRCI35* was carried out in cold sensitive tobacco (*N. tabacum*). The *CbRCI35* expression level was examined in transgenic tobacco lines. Two individual *CbRCI35-ox* lines (L1 and L4) exhibited high transcript levels and were therefore selected for subsequent experiments (Figure 5A). Compared with wild type (WT) and empty vector transformants (EV), L1 and L4 plants showed higher capacity of cold acclimation. WT and EV seedlings both had wilting leaves while L1 and L4 showed a robust status after 1 h of -4°C exposure followed by 2 days of recovery at 22°C (**Figure 5B**). Corresponding physiological analyses were performed using four indices: electrolyte leakage, malondialdehyde (MDA) content, relative water content, and glucose content – all representative indicators of cellular damage under low temperatures (Zhou et al., 2014). After freezing application, relative water contents and glucose content in L1 and L4 were much higher than that of the controls, indicating the protection of cellular water and bioactive components in transgenic plants. In addition, two *CbRCI35-ox* lines showed significantly lower electrolyte leakage and less MDA accumulation under both chilling and freezing stress, which was a symbol of the protection of plasma membrane integrity (**Figure 5C**). These phenotypically demonstrated that *CbRCI35* overexpression increased freezing resistance in tobacco.

**FIGURE 6:**
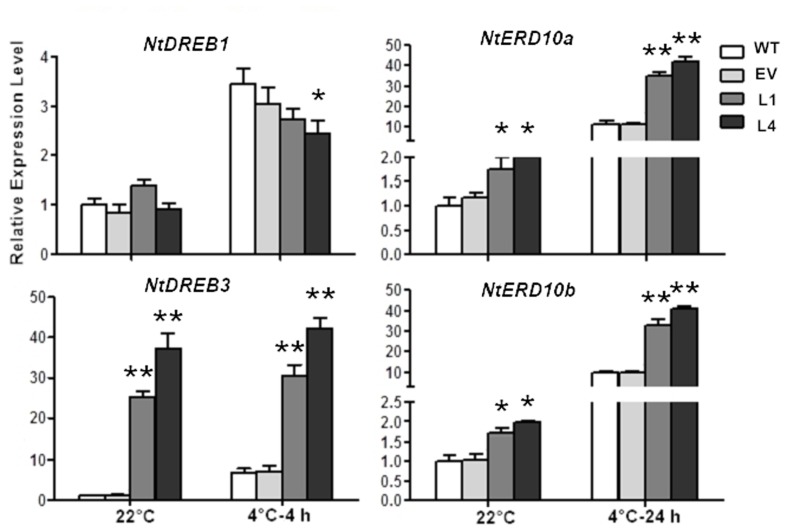
***CbRCI35* activated cold responsive genes in tobacco plants.** The relative expression of *NtDREB1. NtDREB3. NtERD10a*, and *NtERD10b* in *CbRCI35-ox* tobacco plants before and after cold treatments. The *NtActin* gene was performed as an internal control. Data are means ± SD (*n* = 3, ^∗^*P* < 0.05, ^∗∗^*P* < 0.01).

Moreover, the impact of *CbRCI35* on endogenous cold responsive signaling genes in tobacco was investigated. *NtDREB1* and *NtDREB3* belong to CBF/DREB transcription factor family, a group of key cold responsive regulators (Catala et al., 2003; Zhou et al., 2012). *NtERD10a* and *NtERD10b* are downstream CBF/DREB regulons modulated by DREBs (Kasuga et al., 2004). In normal temperatures, *NtDREB3* of L1 and L4 exhibited significantly higher transcript levels than WT or EV. *NtERD10a* as well as *NtERD10b* were also upregulated (**Figure 6**). During treatment in 4°C, *NtDREB3* expression levels of transformants were still much higher than the controls, although they remained similar compared to *NtDREB3* expression at 22°C. *NtERD10a* and *NtERD10b* were remarkably induced by cold and showed much higher transcripts in L1 and L4. *NtDREB1* did not exhibit obviously different expression levels from WT and EV. It can be concluded that *CbRCI35* positively regulated *NtDREB3. NtERD10a*, and *NtERD10b* expression, and conferred enhanced cold induction of these two *ERD10* genes in cold temperature.

### Overexpression of *CbRCI35* Modulated ROS Homeostasis and Altered ROS Metabolic Gene Expression in Tobacco

**FIGURE 7:**
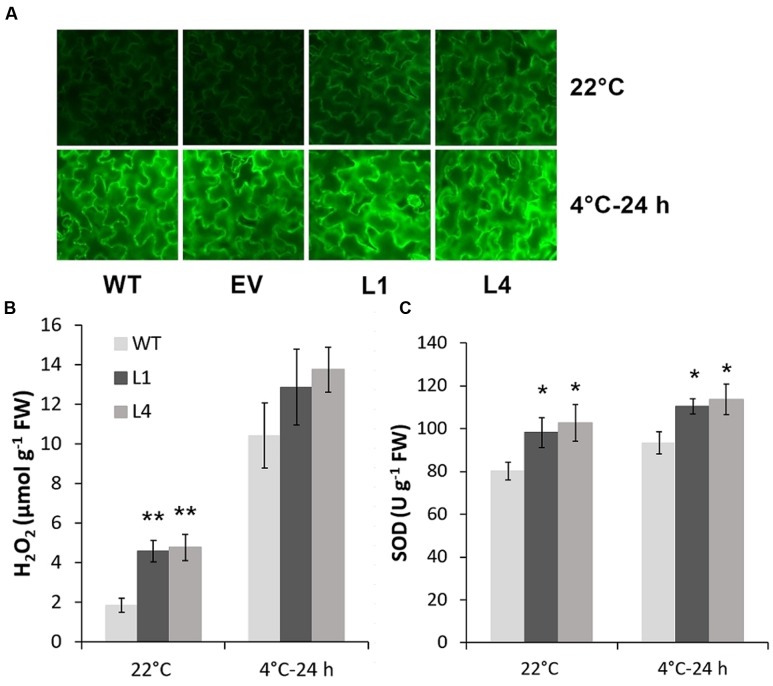
***CbRCI35* elevated tobacco ROS level in normal condition and increased SOD activity after chilling treatment.** **(A)** ROS levels detected by Dichloro-dihydro-fluorescein diacetate (DCFH-DA) assay in 4-week-old tobacco leaf disks with or without 4°C treatment. H_2_O_2_ contents and SOD activity in tobacco leaves of WT and *CbRCI35-ox* lines (L1 and L4) were exhibited in **(B)** and **(C)**, respectively. Error bars indicate SD (*n* = 3, ^∗^*P* < 0.05, ^∗∗^*P* < 0.01).

**FIGURE 8:**
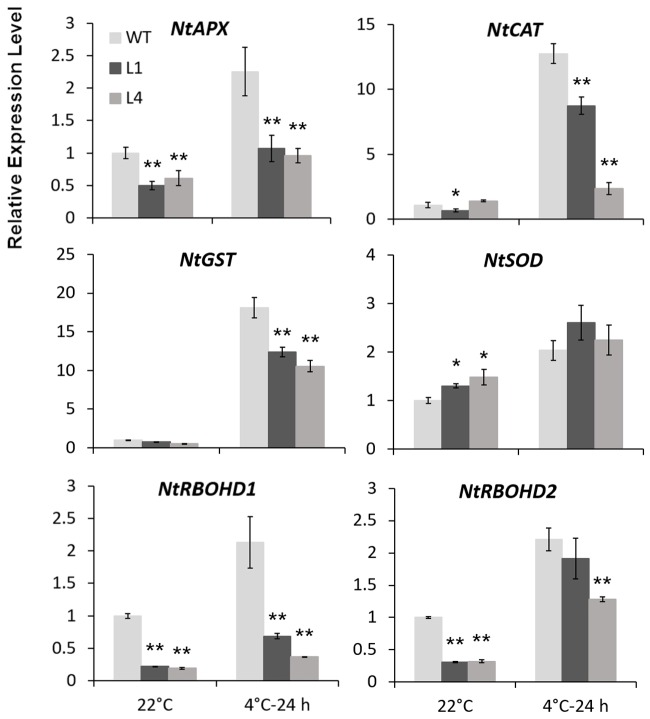
***CbRCI35* altered ROS homeostasis related genes expression.** Relative expression levels of ROS scavenging genes including *NtAPX. NtCAT. NtGST*, and *NtSOD*, as well as ROS biosynthesis genes *NtRBOHD1* and *NtRBOHD2* in *CbRCI35-ox* tobacco plants with or without 4°C treatment. The *NtActin* gene was performed as an internal control. Expression levels were relative to that of WT plants. Data are means ± SD (*n* = 3, ^∗^*P* < 0.05, ^∗∗^*P* < 0.01).

Predicted as a peroxidase, CbRCI35 is supposed to regulate ROS levels in plants. Using the dichloro-dihydro-fluorescein diacetate (DCFH-DA) assay, we verified that two *CbRCI35-ox* tobacco lines exhibited moderately elevated ROS levels before exposure to cold temperatures compared to WT plants. After exposure to 4°C for 24 h, the ROS levels in each tobacco were similar (**Figure 7A**). As a representative component of ROS (Slesak et al., 2007), H_2_O_2_ showed content changes that were in line with DCFH-DA results (**Figure 7B**). We also detected the activity of SOD, a primary antioxidant enzyme in plants (Price et al., 1994). In both normal and chilling conditions, *CbRCI35-ox* tobacco showed higher SOD activity than WT, which can contribute to the protection of cell structure (**Figure 7C**). The increase of ROS at the basal level together with the stronger SOD activity after cold exposure indicated the effects of *CbRCI35* in plant ROS regulation and cell protection. We further examined the expression of ROS metabolic genes in transgenic tobacco. Intriguingly, ROS scavenging and biosynthesis genes were both disrupted (**Figure 8**). In 22°C, transcripts of a key ROS scavenging gene *NtAPX* (Yan et al., 2014) were significantly lower in *CbRCI35-ox* plants, while that of *NtSOD*, another ROS detoxifying gene (Liu et al., 2016), was higher than controls. After 24 h of 4°C application, expression levels of the ROS scavenging genes *NtAPX. NtCAT*, and *NtGST* were noticeably lower in *CbRCI35-ox* plants. Meanwhile, ROS production modulators *NtRBOHD1* and *NtRBOHD2* were downregulated by *CbRCI35* under normal temperature, and the cold induction of *NtRBOHD1* was also significantly blocked in *CbRCI35-ox* tobacco (**Figure 8**). Taken cumulatively, these revealed that the overexpression of *CbRCI35* regulates ROS homeostasis in tobacco.

## Discussion

The *RCI* genes are a group of cold responsive genes identified using *Arabidopsis* cDNA library screening (Jarillo et al., 1994). These genes consist of several different categories. Unlike *AtRCI1A/B* or *AtRCI2A/B. AtRCI3* is a type III peroxidase gene in *Arabidopsis* (Medina et al., 2001; Sivankalyani et al., 2015). With a high similarity to *AtRCI3. CbRCI35* also shows an obvious cold-inducible expression pattern and high transcription level in roots (**Figures 1A,B**). Interestingly, these two genes behave differently in multiple aspects. During cold response, *AtRCI3* transcription is gradually elevated and attained the maximal level after 24 h of 4°C exposure (Llorente et al., 2002). *CbRCI35* responds to the same temperature with a peak of expression levels at 8 h after treatment and returned to a low level at the 24 h time point (**Figure 1B**), indicating its faster activation and potential function in the earlier stage of response to low temperature. For organ dependent expression, *AtRCI3* shows a root specific transcription while *CbRCI35* has a high expression level in roots but can also be induced in leaves and stems (**Figure 1B**). Moreover, *AtRCI3* is expressed not only in the cortex but also in the stele (Llorente et al., 2002), while *CbRCI35* transcription is restricted to the cortex in roots (**Figure 3C**). These data reveal the diverse expressional regulation of type III peroxidase genes from different plant species. Coordinately, the AtRCI3 protein is detected in the endoplasmic reticulum (ER) and can be secreted to the cell wall (Kim et al., 2010), while the CbRCI35 protein is localized in the cytoplasm (**Figures 4B,C**). The distinct transcription pattern and protein localization implies that *CbRCI35* from hardy plant species of *C. bursa-pastoris* may have unique functions distinct from *AtRCI3*. Our work provides a novel insight into the role of AtRCI3-like type III peroxidase in regulating cold tolerance of plants.

Using a cold sensitive species of tobacco, we characterized the molecular function of *CbRCI35* and observed the moderately increased ROS level in transgenic plants at the normal temperature (**Figures 7A,B**). ROS production and scavenging plays a key role in plant cold acclimation (Del Rio, 2015). Plenty of regulators of ROS homeostasis jointly contribute to the control of ROS level and signal transduction (Mangano et al., 2016). Given the widely reported ROS scavengers during stress response in plants, few positive modulators of cold resistance that can augment ROS accumulation have been documented. In our case, overexpression of *CbRCI35* participates in the increase of ROS under normal conditions, which is similar to *AtRCI3* (Kim et al., 2010). The representative well-known genes regulating ROS biosynthesis (*NtRBOHD1* and *NtRBOHD2*) and scavenging (*NtAPX* and *NtCAT*) were repressed, while *NtSOD* expression was slightly enhanced in *CbRCI35-ox* seedlings (**Figure 8**), suggesting that *CbRCI35* may contribute to ROS accumulation through RBOHD1/2-independent pathways in tobacco. The increase of *NtSOD* transcripts can be due to a feedback activation mechanism. In response to chilling treatment, most of these genes were negatively regulated in transgenic tobacco compared with WT control (**Figure 8**). As a result, the ROS level was similar to the control (**Figures 7A,B**), revealing that overexpression of *CbRCI35* reformed the transcriptional control of ROS metabolic genes to facilitate the homeostatic mechanism of ROS. In addition, the SOD activity was higher in transgenic plants in both the warm and chilling conditions, indicating that *CbRCI35* might participate in the protection of bioactive enzymes under cold stress. Although the total ROS level was not lowered, the *CbRCI35-ox* tobacco plants showed alleviated membrane damage suggested by MDA content and electrolyte leakage in chilling temperatures (**Figures 7A,B**). Together with the significantly increased freezing resistance caused by *CbRCI35*, it can be concluded that *CbRCI35* enhanced plant cold acclimation through controlling the ROS homeostasis and activating downstream cold responsive genes. Further, no growth retardation was observed in *CbRCI35-ox* transgenic plants, demonstrating the broad prospects of *CbRCI35* application in plant breeding for crop improvements.

## Author Contributions

JL, MZ, and WL were responsible for the overall experimental design and conduct of the experiments. MZ performed the cold induction test, promoter isolation, and tobacco transformation. JL and MZ took the lead on manuscript development. WL and YZ conducted the gene expressional analysis, promoter activity investigation, protein localization detection, and transgenic tobacco characterization. PL and XY contributed to experimental design and data analyses. All authors read and approved the final manuscript.

## Conflict of Interest Statement

The authors declare that the research was conducted in the absence of any commercial or financial relationships that could be construed as a potential conflict of interest.

## Footnotes

1 http://bioinformatics.psb.ugent.be/webtools/plantcare/html/

2 http://bioinformatics.psb.ugent.be/webtools/plantcare/html/

3 http://www.csbio.sjtu.edu.cn/bioinf/plant-multi/
